# Through thick and thin: nursing resilience as an adaptive psychological mechanism in the context of professional integration

**DOI:** 10.1186/s12912-025-04003-x

**Published:** 2025-11-04

**Authors:** Camille Gagnon-Béland, Stéphanie Austin, Liette St-Pierre

**Affiliations:** 1 Département des sciences infirmières, Groupe de recherche sur la motivation et le mieux-être, Université du Québec à Trois-Rivières, 3351 Bd des Forges, Trois-Rivières, QC G8Z 4M3 Canada; 2 Département de gestion des ressources humaines, Groupe de recherche sur la motivation et le mieux-être, École de gestion, Université du Québec à Trois-Rivières, 3351 Bd des Forges, Trois-Rivières, QC G8Z 4M3 Canada; 3 Département des sciences infirmières, Chaire interdisciplinaire de recherche et d’intervention dans les services de santé, Université du Québec à Trois-Rivières, 3351 Bd des Forges, Trois-Rivières, QC G8Z 4M3 Canada

**Keywords:** Resilience, Basic psychological needs, Quality of interpersonal relationships, Occupational health, Occupational functioning, Mediation analyses

## Abstract

**Background:**

Based on the theoretical framework of self-determination theory, this study examines the links between the quality of interpersonal relationships and occupational health (vigor and emotional exhaustion), as well as the occupational functioning (affective commitment and turnover intentions) of new nurses, considering the potential role of psychological resources (satisfaction of basic psychological needs: autonomy, competence and relatedness; resilience) as intermediate coping mechanisms.

**Method:**

The sample for this cross-sectional study comprises 273 Quebec new nurses (≤ 2 years of experience). Using the MPlus 8.8 software, a mediation analysis was performed to test the proposed adaptation model.

**Results:**

The quality of interpersonal relationships at the beginning of a nursing career is linked to the satisfaction of psychological needs at work. In this regard, our results indicate that it is through the perception of competence at work that the quality of interpersonal relationships is associated with resilience. In turn, new nurses’ resilience is related to occupational health (positive and significant link with vigor, negative and significant link with emotional exhaustion). In terms of the psychological resources that potentially intervene in the relationship between the quality of interpersonal relationships and the occupational functioning, our results suggest that psychological needs could play a partial mediating role. Indeed, direct and significant links are observed between the perception of competence and the occupational turnover intentions (β = − 0.226, *p* = .029), as well as between the perception of autonomy and affective commitment to the nursing profession (β = 0.240, *p* = .026).

**Conclusion and implications:**

Positive relationships at work are a lever for professional adaptation for new nurses since they lead to the establishment of important psychological resources (competence and resilience) for their occupational health and occupational functioning. Health care institutions are therefore encouraged to strengthen harmonious, rewarding, satisfying and trusting relationships in their environments. In addition, they would benefit from directing the new nurse to the importance of their psychological resources, given their positive associations with the health and functioning indicators measured in this study. The implications of the study are discussed in light of the most recent literature on the adaptation of new nurses.

**Trial registration:**

Not applicable.

## Introduction

The beginning of a nursing career is difficult for many new nurses due to daily stress of practice for which they are poorly prepared [1]. Yet, the difficulties encountered at this pivotal moment in their professional journey may shape their career path. This is why several studies have been carried out so far on the adaptation of new nurses to work. It is now clear that the adaptation of new nurses to the job depends largely on the adequacy between the demands of their work, the resources available to them, including those needed to meet the needs and expectations of their environment and practice. However, few studies have been conducted to date to examine the adjustment of new nurses in terms of both their occupational functioning and their occupational health. In this regard, the concomitant examination of the psychosocial and organizational determinants of the adaptation of new nurses at the beginning of their careers is insufficient [2].

Increasingly, fostering a culture that supports health and optimal functioning at work is seen as a promising approach to addressing work-related stressors and their associated challenges [3]. The workplace resources deployed to support the new nurse in their adaptation are a testament to this culture [2]. They generally have a positive influence on the experience of new nurses, in particular by promoting the development of the quality of interpersonal relationships [4, 5]. By contributing to the phenomenon of socialization at the beginning of their careers, they predispose new nurses to adopt norms, values and behaviours that are more adapted to their new work environment [2].

According to some authors, these workplace resources are particularly favourable to professional adaptation when they support the basic psychological needs for autonomy, competence and relatedness. Rooted in self-determination theory (SDT; [6]), the basic psychological needs theory is one of its mini-theories. SDT posits that fulfilling these basic needs is essential for sustaining psychological interest, development, and well-being. These needs are considered fundamental because they support adaptation and optimal functioning across all environments. The need for autonomy refers to the individual’s need to act with a sense of ownership of his/her behaviours and to feel psychologically free to act [7]. For the new nurse, it is a question of acting while controlling his/her actions rather than seeing them dictated by external forces or demands [8]. For its part, the need for competence concerns the individual’s judgment in relation to his/her abilities. A good example is the new nurse who feels empowered to perform their tasks with a positive outcome. Relatedness, on the other hand, refers to the appreciation of the bonds established or felt with others [6, 7, 9]. This is, for example, the new nurse who feels they benefit from positive and meaningful relationships with key individuals in their work environment.

Importantly, the work resulting from SDT converges on the idea that, when supported, the basic psychological needs constitute important personal resources for self-determination [6], i.e., the possibility of acting according to one’s interests, values and objectives. As a result, these are important levers of adaptation [10], while also fostering the development of other psychological assets that are essential for successful integration into the workplace.

An example of a psychological resource that has been widely documented in recent years is resilience [11, 12]. The state of the literature is clear that resilience is a psychological resource necessary for occupational health and functioning [13], and especially for new nurses at the beginning of their careers. When contextualized to professional integration, resilience can be treated as *situational*, manifesting itself temporarily when the new nurse is, for example, exposed to a stressful situation. Recently, a new definition of nursing resilience was proposed through an integrative review of the literature, focusing on the context of professional integration during the first two years of nursing practice. It is as follows: “Nursing resilience at the beginning of a career is a psychological resource that can be actively developed to strengthen new nurses’ psychological, physical, and social well-being, while also reducing states of distress. This is achieved through positive capacities such as adaptability, flexibility, optimism, effective problem-solving, and the confidence to adequately cope with the stressors associated with early-career nursing – whether these stem from the nature of the work itself, the practice settings, or their management.” [14] (p. 13). Nursing resilience therefore reflects what new nurses actually experience in situ [15]. This perspective thus helps to understand how nursing resilience can act as a personal and adaptive resource at the beginning of a career [16], in the face of occasional stressors of nursing work.

However, despite the interest in workplace resilience in the scientific literature, the body of research remains relatively silent on its antecedents and on the psychological mechanisms that come into play in explaining its effects on occupational health and functioning. Therefore, it seems essential to examine how resilience as a personal resource allows new nurses to better adapt and function well at the beginning of their careers. It is equally important to examine elements of the work context, such as the quality of relationships, and other personal resources of new nurses, such as basic psychological needs at work, that predispose to nursing resilience and promote adaptation in the early stages of a nursing career.

## Methods

### Aims

**Fig. 1:**
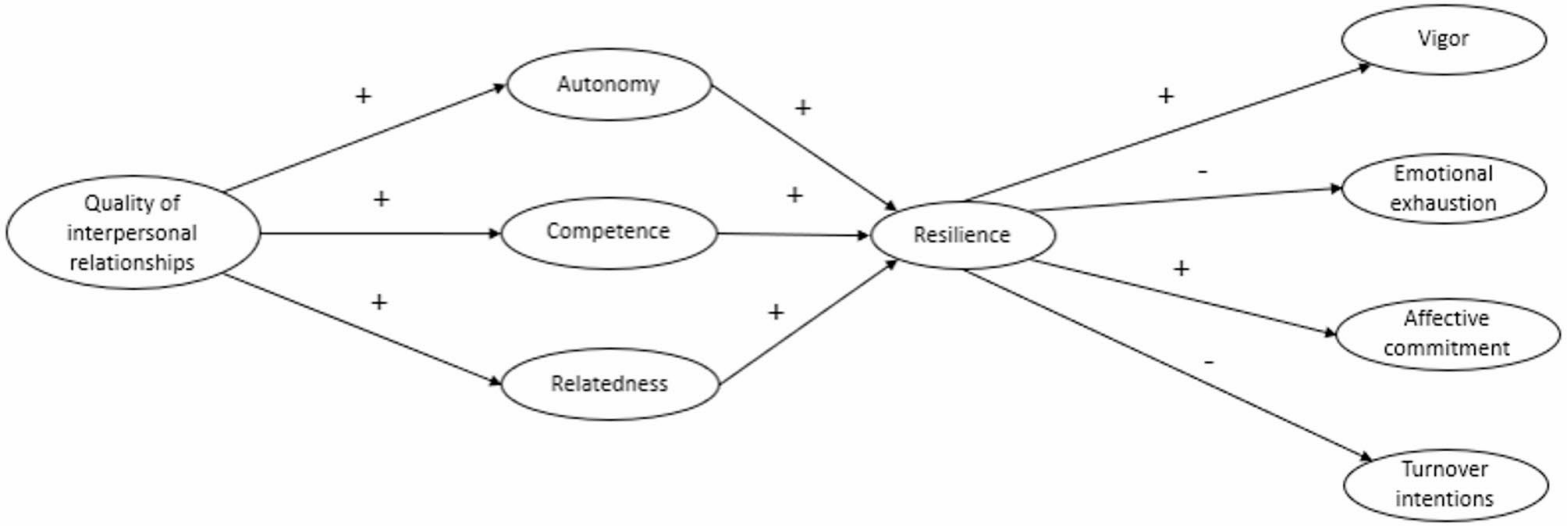
The proposed theoretical model

According to a sequence of “Context→Needs→Resilience→Adaptation” type, our study examines the direct and indirect links between workplace resources (perception of the quality of interpersonal relationships with the referent at work) and personal psychological resources of new nurses (basic psychological needs for autonomy, competence and relatedness; nursing resilience) on their occupational health and functioning at the beginning of their careers. More specifically, the conceptual model developed proposes that the psychological needs and resilience of new nurses act as mediators in the relationship between workplace resources and the adaptation of new nurses, both in terms of occupational health and functioning (see the conceptual model of work adaptation of new nurses (Fig. 1). Highlighting the links between the concepts of interest will provide a more in-depth insight into the resources or personal characteristics of new nurses and with regard to the professional integration experience. To this end, nursing resilience alone cannot facilitate professional integration [17]. And as far as we know, few studies have demonstrated the links between psychological needs and career adjustment [18]. In terms of health, two indicators that are widely documented in the literature as so-called work-specific experiences are examined: vigor at work (reserve of emotional energy invested at work [19]) and emotional exhaustion (feeling of not having the energy required to do the job, depriving them of the energy required to adapt at the beginning of their careers [20, 21]). In terms of occupational functioning, affective commitment (a psychological state that indicates a positive emotional orientation or attachment of the individual to his/her work [22]) is examined, as well as the turnover intentions (conscious desire to leave the profession [23]). Together, these different indicators will highlight the benefits and harms associated with differentiated levels of personal resources and the work of new nurses.

With regard to SDT, interpersonal relationships are considered a resource in the work context that plays a key role in supporting the satisfaction of psychological needs since they offer determinants of motivation, well-being, as well as development opportunities [6]. Following this idea, for many nurses, it is important to contribute to the development of colleagues. These nurses, often identified as a “significant reference person” by new nurses, are instrumental in establishing a healthy work environment (i.e., safe, empowering and supportive [1]). Among other things, the significant referent can play a key role in fostering supportive relationships that are better aligned with the needs of new nurses [24, 25]. Moreover, they are more inclined to perceive high-quality relationships in their work referents [26–28]. Based on these theoretical and practical considerations, we propose the following hypothesis:

#### H1a

The high-quality interpersonal relationships with the significant referent are positively associated with the satisfaction of the need for autonomy.

#### H1b

The high-quality interpersonal relationships with the significant referent are positively associated with the satisfaction of the need for competence.

#### H1c

The high-quality interpersonal relationships with the significant referent are positively associated with the satisfaction of the need for relatedness.

Research confirms the contribution of everyday personal resources to adaptation and occupational functioning. In accordance with SDT, meeting psychological needs leads to higher levels of resilience. One study shows the link between psychological needs taken as a whole, without distinguishing between them, and situational resilience [11], while others have shown the specific link established between the need for competence and resilience (state of resilience [29]; resilience trait [30]), when the latter variable is presented as a mediator. More specifically to the nursing population, the study by Bai and Bai [31] focused on examining a serial mediation process, involving both basic psychological needs (overall measure) and trait resilience. Although specific to the targeted profession, this study does not distinguish between the three psychological needs. However, each of the needs could be distinctly associated with higher or lower levels of resilience, which is important information to know when intervening on elements to better equip new nurses. In addition, it is important to note that the concept of resilience used in this study refers to a trait, rather than a resource that is expressed in the face of occasional stressors of nursing work at the beginning of a career. Nevertheless, there are some studies in other fields and conducted with other populations of workers that have distinctively mobilized psychological needs at work in order to identify the most important need(s) for adaptation to work. In most of these studies, the need for competence emerged as the main determinant of workers’ resilience, regardless of population. Since we distinguish between the three psychological needs in our study in order to broaden the practical scope of our findings, we make the following specific hypothesis:

#### H2a

The psychological need for autonomy is positively associated with nursing resilience.

#### H2b

The psychological need for competence is positively associated with nursing resilience.

#### H2c

The psychological need for relatedness is positively associated with nursing resilience.

Early nursing research identifies that resilience is an important adaptative resource, particularly because it reduces the symptom of emotional exhaustion [32] that is prevalent in the profession. Referring to an emotional tension reaction [33] that is detrimental to new nurses’ occupational health, emotional exhaustion deprives new nurses of the energy needed to adapt early in their careers [20, 21]. Also, research results show that resilience is reflected in new nurses’ engagement and experiences at work [34]. For example, resilience is thought to promote awareness of the benefits of nursing [35]. It is also thought to predispose to work involvement (i.e., a motivational factor characterized by vigor at work [33, 36]), and in return, would decrease the turnover intentions [37]. These impacts are significant concerns for many professions, including nursing. In connection with the results of available studies and contextualized to professional integration, we propose that nursing resilience fosters optimal psychological health in the workplace, reflected in elevated states of well-being and, correspondingly, reduced levels of distress. More specifically:

#### H3a

Nursing resilience is positively associated with the work vigor of new nurses.

#### H3b

Nursing resilience is negatively associated with the emotional exhaustion of new nurses.

As a corollary, we propose that nursing resilience contributes positively to the optimal functioning of new nurses at the beginning of their careers. More specifically:

#### H3c

Nursing resilience is positively associated with affective commitment to the nursing profession.

#### H3d

Nursing resilience is negatively associated with occupational turnover intentions.

The purpose of this study is to examine how high-quality of interpersonal relationships are associated with occupational health and functioning by investigating the satisfaction of psychological needs and nursing resilience. On the theoretical and empirical basis, we propose a model derived from SDT. According to the model, the quality of interpersonal relationships is related to occupational health, which is expressed through vigor and emotional exhaustion, and to occupational functioning, which is manifested by affective commitment and turnover intentions. Also, the model proposes that it be through support for psychological needs and nursing resilience – psychological and adaptive mechanisms – that the quality of interpersonal relationships is linked to occupational health and functioning at the beginning of a nursing career. Together, the research hypotheses will examine the extent to which workplace resources in the early nursing career are associated with new nurses’ personal resources, occupational health and functioning. A potential contribution of this research is therefore the look at basic psychological needs, taken distinctively, namely whether they are all conducive to the career adjustment of new nurses [18], and how? We argue that it is through nursing resilience that psychological needs, strengthened by strong early-career relationships, can explain their adaptation, both in terms of health and functioning. We therefore believe that this study is innovative because it will allow us to better understand the potential mediating role of psychological needs and nursing resilience in the relationship between the perception of the quality of their relationships at work with their significant referent and their adaptation to work. Arguing that the quality of this relationship enables new nurses to fully mobilize their motivational resources at a critical point in their career path, and that in turn, these resources enhance their resilience, it is interesting to examine to what extent and how they translate into adaptive attitudes and behaviours at work. Thus, based on the SDT as well as on the results of studies presented above, we propose that:

#### H4

Personal resources (needs for autonomy, competence and relatedness at work, and resilience) mediate the link between the quality of interpersonal relationships with the significant referent at work and the adaptation of new nurses during the early stages of their careers.

### Desing and analytical approach

This study is quantitative, descriptive-correlational, and cross-sectional. Bootstrapping analyses were used to enhance parameter estimation within the framework of structural equation modeling. The Mplus 8.8 software was used to estimate the model.

### Sample

The data used for this study was collected over a one-year period, from August 2023 to September 2024. The participants were recruited online, as well as in person in university classrooms. The participants were invited to complete an online or paper questionnaire, in French. Their participation was voluntary and confidential; they were assigned a unique identifier to ensure confidentiality and anonymity. Out of a sample of 298 new nurses with less than two years of career experience in health and social service institutions in the province of Quebec (Canada), 273 answered all the questions of the research questionnaire. On average, the participants were 26.77 years old (SD = 7.815) and were female (90.6%). This demographic profile is similar to provincial statistics on the next generation of nurses. All of them were trained as nurses, ensuring that they were at least candidates for the nursing profession. Nearly half of the participants.es (49.8%) had a formal referent in the workplace, compared to 50.2% without a formal referent. Also asked about their informal referent, 63.5% of participants indicated that they had one, while 36.5% indicated that they did not have an informal referent. Socio-demographic data are detailed in Table 1.

In addition, a high proportion of new nurses in our sample (73.4%) had a stress level above or equal to the average ($\bar{\text{X}}$ = 3.24 [SD = 1.069]; [scale: 1 to 5]). A slightly above-average resilience rate ($\bar{\text{X}}$ = 3.67 [SD = 0.559]; [scale: 1 to 5]) was found, which is similar to those documented (14).

**Table 1:** Socio-demographic characteristics

	Mean	SD
Age (*n* = 290)	26.77	7.81
	Nb	%
Gender (*n* = 298)		
• Female	218	90.6
• Male	23	9.4
As a couple (*n* = 296)	191	64.1
With child(ren) (*n* = 298)	68	22.8
Support (*n* = 296)	285	95.6
Training (*n* = 297)		
• Collegiate institute	238	79.9
• University	59	19.8
Administrative region of Quebec (*n* = 291)		
1. Abitibi-Témiscamingue	5	1.7
2. Bas-Saint-Laurent	3	1.0
3. Capital-Nationale	13	4.4
4. Centre-du-Québec	38	12.8
5. Chaudière-Appalaches	4	1.3
6. Côte-Nord	3	1.0
7. Estrie	45	15.1
8. Laval	6	2.0
9. Lanaudière	10	3.4
10. Laurentides	8	2.7
11. Mauricie	57	19.1
12. Montérégie	41	13.3
13. Montréal	51	17.1
14. Outaouais	4	1.3
15. Saguenay-Lac-Saint-Jean	3	1.0
Care unit (*n* = 296)		
• Medicine-Surgery	146	49.0
• Geriatrics/Long-term care	34	11.4
• Emergency care	23	7.7
• Mother-child unit/Postnatal care	23	7.7
• Paediatrics	15	5.0
• Other (e.g., delivery room, specialized and ultra-specialized care, oncology, primary care, intensive and neonatal care, day surgery, physical disability, etc.)	55	18.4
Working hours (*n* = 297)		
• Full time	100	33.6
• Partial time	197	66.1
Shift (*n* = 297)		
• Day	55	18.5
• Evening	58	19.5
• Night	35	11.7
• Day/Evening	116	38.9
• Day/Night	17	5.7
• Evening/Night	16	5.4

Note. *n* = 298. Some missing data explains why the totals are less than 100%

### Measuring instruments

The data for this study were collected using a set of validated instruments, whose psychometric qualities have been established in previous research [38, 39]. These instruments were embedded within a broader questionnaire originally developed as part of the first author’s doctoral dissertation. The standardized measurement tools used in this study are as follows:

#### Quality of interpersonal relations

The Quality of Interpersonal Relationships Scale (QIRS; [40]-4 items; α = 0.951) was used to measure the extent to which the relationships maintained with the main referent at work (formal or informal) are harmonious, rewarding, satisfying and imbued with trust. Each statement was rated on a four-point scale, ranging from 0 (not at all) to 4 (extremely). An example of an element is: “My relationship with my referent is harmonious”.

#### Basic psychological needs

The Psychological Need States at Work-Scale (PNSW-S; [41]- 12 items, α = 0.927) was used to measure the satisfaction of basic psychological needs of autonomy (3 items; α = 0.871; e.g., “I feel free to make choices with regards to the way I work”); competence (3 items; α = 0.898; e.g., “I feel I am capable”); and relatedness (6 items; α = 0.942; e.g., “I feel supported”). Each statement was rated on a seven-point scale ranging from 1 (strongly disagree) to 7 (strongly agree).

#### Resilience

The Brief Resilient Coping Scale (BRSC; [42]-4 items; α = 0.579) was used to measure the tendency to cope with stress in an adaptive manner. Each statement was rated on a five-point scale ranging from 1 (does not describe me at all) to 5 (describes me very well). An example of an item is, “Regardless of what happens to me at work, I believe I can control my reaction.”

#### Vigor at work

The Ultra-Short Measure for Work Engagement ([19]-3 items; α = 0.840) derived from the Utrecht Work Engagement Scale (UWES-9; [43]) was used to assess high levels of energy at work. Each statement was rated on a seven-point scale ranging from 1 (never) to 7 (every day). An example of an item is: “At my work, I feel bursting with energy.”

#### Emotional exhaustion

The short subscale ([44]-5 items, α = 0.907) derived from the Maslach Burnout Inventory - General Survey (MBI-GS-22; [45]) was used to assess work-related feelings of emotional exhaustion. Each statement was rated on a seven-point scale ranging from 1 (never) to 7 (every day). An example of an item is, “I feel emotionally drained from my work.”

#### Affective commitment

The affective component measure of the Occupational Commitment Scales ([46]- 3 items, α = 0.898) was used to assess the prediction of occupational activity. Each statement was rated on a seven-point scale ranging from 1 (strongly disagree) to 7 (strongly agree). An example of an item is: “I feel emotionally attached to my occupation.”

#### Occupational turnover intentions

The French version ([47]- 5 items, α = 0.909) of the adapted scale [48] was used to measure occupational turnover intentions. Each statement was rated on a seven-point scale ranging from 1 (strongly disagree) to 7 (strongly agree). An example of an item is: “I’m thinking about quitting my current care facility.”

## Results

### Descriptive and correlational statistics

The values of the means, standard deviations and correlations for each global scale are presented (see Table 2). The reliability of the measurements was established by Cronbach’s alpha coefficient. Values equal to or greater than 0.70 are considered satisfactory [49].

**Table 2:** Means, standard deviations and correlations between variables

Variables	Mean	SD	1	2	3	4	5	6	7	8	9
1. Quality of interpersonal relationships	2.83	0.971	(0.951)								
2. Autonomy	4.00	1.253	0.434	(0.871)							
3. Competence	4.78	1.041	0.284	0.390	(0.898)						
4. Relatedness	4.14	1.361	0.589	0.617	0.475	(0.942)					
5. Resilience	3.67	0.559	0.210	0.254	0.422	0.276	(0.579)				
6. Vigor	4.24	1.103	0.318	0.375	0.455	0.408	0.482	(0.840)			
7. Emotional exhaustion	3.97	1.389	− 0.251	− 0.270	− 0.300	− 0.329	− 0.385	− 0.578	(0.907)		
8. Affective commitment	5.11	1.256	0.270	0.343	0.355	0.303	0.210	0.390	− 0.149	(0.898)	
9. Turnover intentions	1.94	1.337	− 0.248	− 0.332	− 0.400	− 0.318	− 0.294	− 0.445	0.543	− 0.319	(0.909)

Note. Correlations of 0.10-0.20 are significant at *p* < .05. Correlations of 0.20 and higher are significant at *p* < .001. Cronbach’s alpha in brackets

### Statistical analyses

Structural equation modelling aims to designate assumptions about the means, variances, and covariances of the observed variables in terms of a smaller number of structural parameters defined by an assumed underlying conceptual model. Specifically, this modelling aims to (a) show that the null hypothesis (the theoretical model with all its paths) is not significant, but that specified paths are adequate, and (b) state indices of overall adequacy through different measures showing other adequacy [50].

### Preliminary analysis

The model was tested with standardized coefficients obtained by estimating the maximum likelihood. The fit of the model was assessed using five fit indices: the ratio of a chi-square statistic to its respective degrees of freedom (χ2/df), the Root Mean Square Error of Approximation (RMSEA) and its confidence interval (CI), the Comparative Fit Index (CFI), the Tucker-Lewis Index (TLI), and the Standardized Root Mean Square Residual (SRMR). The adjustment indices obtained for this model – comprising the three factors of satisfaction of needs taken separately – were as follows: χ2/d = 940.057*(558), RMSEA = 0.050 [0.044; 0.056], CFI = 0.932, TLI = 0.924 and SRMR = 0.049. After analyzing the overall fit, we agreed that this model meets the criteria for descriptive measures of fit. The results of the proposed model showed no negative variance, indicating that the model did not violate the identification rules. According to Kline [51], a chi-square test with a value equal to or less than 3 suggests a good fit of the model. Here, by dividing the degrees of freedom with the chi-square, we get 1.685, which shows that the model is adequate. The other indices of the quality of the model fit were also within the acceptable range. Indeed, we see favourable statistics: the RMSEA value of 0.050, which indicated an excellent fit and was within the desired range of 0.05 to 0.08 to represent a reasonable approximation error, the CFI and TLI values, of 0.932 and 0.924, respectively, were slightly above the required threshold of 0.90 and above and finally, the SRMR value of 0.049 was well below the desired value of less than 0.08 [50].

Next, the measurement model was tested, in which the observed variables were loaded onto their respective latent factor. To confirm the multidimensional nature of the concept of psychological needs satisfaction, a global model was also tested, in which the three psychological needs were considered jointly rather than being treated as distinct first-order factors (χ2/df = 978.023*(571), RMSEA = 0.051 [0.046; 0.056], CFI = 0.928, TLI = 0.920 and SRMR = 0.060). Compared to this global satisfaction model, we chose to represent need satisfaction through these three distinct psychological needs. In this way, we were able to support the presence of well-differentiated relationships between each specific need and thus, the distinctive nature of each need [52]. Figure 1 illustrates the proposed theoretical model.

### Model testing

**Fig. 2:**
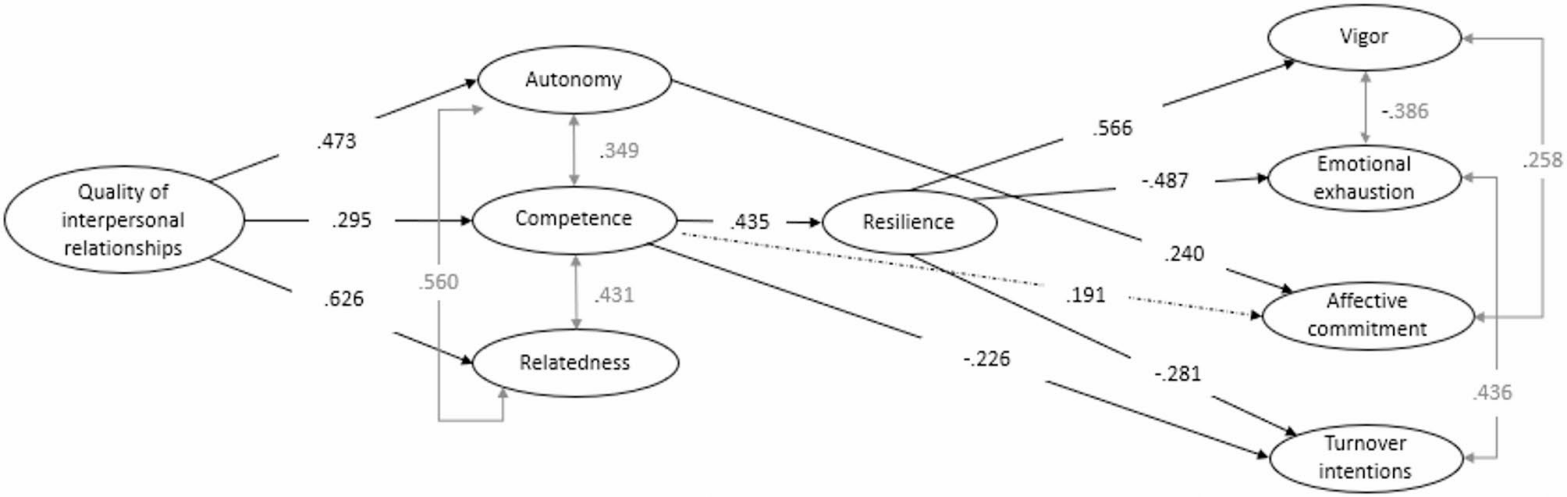
The final model. Note. Solid lines represent significant relationships (*p* < .05). The dashed line shows a link with a value of *p* = .050

A structural equation model was tested to test the assumptions (see Fig. 2). This model includes an indirect link between the quality of interpersonal relationships and both positive and negative indicators of new nurses’ occupational health, through the satisfaction of psychological needs and nursing resilience. For each psychological need, a covariance was allowed. For the indicators of vigor and emotional exhaustion, covariance was allowed, as well as for the indicators of affective commitment and turnover intentions. The model fit particularly well with the data (χ2/df = 940.057*(558), RMSEA = 0.050 [0.044; 0.056], CFI = 0.932, TLI = 0.924 and SRMR = 0.049). High-quality relationships predict the three basic needs, supporting hypotheses 1a, 1b, and 1c. Also, only the need for competence, when satisfied, is related to nursing resilience, in accordance with hypothesis 2b. However, hypotheses 2a and 2c have not been confirmed. Resilience, on the other hand, has a positive effect on vigor in accordance with hypothesis 3a, but does not have a significant effect on affective commitment, rejecting hypothesis 3c. In addition, resilience negatively affects negative occupational health and functioning indicators (emotional exhaustion and occupational turnover intentions) in accordance with hypotheses 3b and 3d. In addition, the model demonstrated that the need for competence negatively affects on turnover intentions, while the need for autonomy is associated with affective commitment. Also, we are able to observe a trend in the prediction of affective commitment through the need for competence.

To more formally test the mediation pathways proposed in the final model (see Table 3), 95% confidence intervals were calculated from 5000 Bootstrap samples. This number of replications is important because it represents efficient and accurate resampling and thus improves estimates [53]. The standardized results show that the quality of interpersonal relationships has a partial indirect effect on resilience through the need for competence. The need for competence has a partial indirect effect on vigor through resilience. The quality of interpersonal relationships has a partial indirect effect on vigor through the need for competence and resilience. The need for competence has a partial indirect effect on emotional exhaustion through resilience. The quality of interpersonal relationships has a partial indirect effect on emotional exhaustion through the need for competence and resilience. The need for competence has a partial indirect effect on the turnover intentions through resilience. The quality of interpersonal relationships has a partial indirect effect on the turnover intention through the need for competence as well as through the need for competence and resilience. These results support, in part, hypothesis 4. Based on these results, we find that the quality of interpersonal relationships has a partial indirect effect on affective commitment through the need for autonomy. In addition, we see a trend towards the effect of the quality of interpersonal relationships on affective commitment through competence.

**Table 3:** Statistical mediation of interpersonal relations and occupational outcomes via psychological needs and resilience

				95% CI	
Predictor	Mediating variable	Outcome	Estimate	Lower	Upper
Quality of interpersonal relationships Quality of interpersonal relationships Quality of interpersonal relationships Quality of interpersonal relationships Quality of interpersonal relationships Competence Competence Competence Quality of interpersonal relationships Quality of interpersonal relationships	Competence Competence + Resilience Competence + Resilience Competence Competence + Resilience Resilience Resilience Resilience Autonomy Competence	Resilience Vigor Emotional exhaustion Turnover intentions Turnover intentions Vigor Emotional exhaustion Turnover intentions Affective commitment Affective commitment	0.129 0.073 − 0.063 − 0.067 − 0.036 0.246 − 0.212 − 0.122 0.114 0.057	0.061 0.032 − 0.148 − 0.151 − 0.093 0.125 − 0.421 − 0.263 0.015 0.003	0.229 0.159 − 0.026 − 0.009 − 0.013 0.451 − 0.097 − 0.044 0.220 0.138

In sum, the results show that the need for competence and nursing resilience act as serial partial mediators, promoting new nurses’ work vigor and protecting them from emotional exhaustion and turnover intention. The need for autonomy, for its part, partially explains the link between the quality of interpersonal relationships and affective commitment. A similar trend is observed through the need for competence. In addition, we do not observe any significant link based on the need for relatedness.

## Discussion

We proposed a derivative model, inspired by the theoretical foundations of SDT, in order to better understand the adaptation of new nurses at the beginning of their careers. The model suggests that high-quality interpersonal relationships enhance occupational health and functioning by fulfilling psychological needs and fostering resilience among nurses. The results of the structural equation modelling support this model. Contextualized to professional integration, the results highlight the proposition that the need for competence and nursing resilience act as serial partial mediators between the quality of interpersonal relationships and occupational health and functioning; in other words, these psychological and adaptive mechanisms promote vigor at work, in addition to protecting new nurses from emotional exhaustion and occupational turnover intentions. Affective commitment to the nursing profession, for its part, is fostered through the need for autonomy. A trend towards the need for competence is also noted. As a result, these motivational and adaptive resources play a particularly important role in promoting the health and optimal functioning of new nurses (more positive and less negative impacts). In some ways, positive working relationships allow new nurses to feel self-motivated at work, to exercise their full potential, and to adopt a more flexible posture in the face of daily challenges [54, 55].

### Theoretical implications

#### Quality of interpersonal relationships and basic psychological needs

The significant referent adopts one of the most influential roles at the beginning of a nursing career. Among other things, the quality of one’s relationships with the new nurse is essential to their occupational health and functioning, by acting on their psychological and professional development. Our model is in agreement with current empirical knowledge [54]. Our results show that the quality of interpersonal relationships with the significant referent at work supports the three psychological needs [6, 7, 9, 56]. Therefore, the quality of relationships can consolidate new nurses’ autonomous motivation [57] despite the stressors of work. Nevertheless, this observation, knowledge remains unclear about the effect of interpersonal relationships at the beginning of a nursing career [58]. The relational aspects of the temporal perspective in relationships with the significant referent, formal or informal, need to be better understood [59]. Thus, the results of our study add to the knowledge by highlighting the significant associations between these variables of interest. In the same line of ideas as Dwyer and Revell (systematic review; [60]), our results suggest that high-quality relationships with a referent are conducive to the creation of the bonds necessary for the development of psychological resources. Through their supportive practices, the referent reinforces autonomy and security towards the development of competencies [61]. Indeed, in his/her relationships, the referent must attach particular importance to the new professional role of the new nurse within his/her new work environment. The referent must also protect the new nurse from an inappropriate workload and thus focus on safety in addition to giving rise to open communication and positive feedback on a regular basis [24]. Also, the competencies of new nurses are conducive to the safety of care and protect the “credibility”. In particular, they contribute to making them independent at work [62] and therefore, more autonomous. Finally, it goes without saying that these opportunities for reinforcement are beneficial to the feeling of relatedness in the relationships that are established. In addition, the role of the referent is to promote a sense of belonging within the work team, so that the new nurse feels like a full member of the team. Conversely, studies that examine lower-quality relationships – which provide feelings of humiliation or inferiority, for example – highlight dependence on early career support, largely because new nurses’ competencies are evolving in a new environment. These relationships usually stem from a perceived lack of competence [63]. Yet, improving workplace health from a well-being perspective is at the heart of health priorities. Following this idea, our results illustrate the contribution of positive supportive practices, which are directed towards states of improvement, development or change affecting workplace well-being.

#### Basic psychological needs and nursing resilience

Another contribution of this study is the examination of nursing resilience as an adaptive psychological mechanism that may contribute to understand the differential role of need satisfaction in predicting occupational health and functioning. The satisfaction of psychological needs makes it possible to internalize professional experiences [64]. Based on SDT, we offer a better understanding of *how* (responses to needs) and *why* new nurses are resilient in the context of professional integration. By measuring psychological needs separately, our results add to the knowledge by specifying the role of each psychological need in the adaptive psychological mechanism of nursing resilience in the context of professional integration.

In addition, it is not surprising to us to note that at the beginning of a career, the need for competence is *the one* that is linked nursing resilience. This result leads us to consider that the satisfaction of new nurses’ need for competence is an integral part of their resilience process, unlike the other two psychological needs. This represents a distinctive contribution to the existing body of knowledge on resilience, particularly within the context of early-career nursing. Building on this idea, Sonmez et al. [18] have shown that competence is the need that most predicts career adaptation. In particular, the adaptation of new nurses is at the heart of the concerns; this is closely linked to the health network’s ability to fulfill its main long-term mission – the provision of quality care and services – which remains a central priority of Québec’s Ministère de la Santé et des Services sociaux (MSSS). Following this idea, competence is the main goal of nursing practice [62]. Indeed, structured education and training programs in mentoring or supervisory activities focus on the proactive development of clinical reasoning competencies [24], essentially because the clinical dimension is one of the main strengths of nurses. For example, nursing education is based on a competency framework [65] that is based on the aggregation of clearly defined objectives, and which represents the vision of the progression of nursing practice. In this context, the perception of acting in accordance with the demands and of being able to achieve the desired results at work takes on its full meaning. Through this feeling, and through his/her competencies in the strict sense, the new nurse contributes to his/her own development and ultimately transforms both his/her own experience and that of the health care to which he/she contributes.

Moreover, several theoretical models support professional integration. For example, the theory of clinical expertise development through stages of learning, *from novice to expert* [66], proposes that new nurses with less than two years of career experience would have the “initial” competencies to recognize and evaluate clinical situations. According to this theory, they present difficulties in analyzing tasks, managing concomitant requests and prioritizing clinical situations and interventions. As a result, they may “not know or be able to do so” [61]. Competence could therefore be the need to focus on to consolidate nursing resilience at the beginning of a career. In addition, this explanation supports the importance of professional development as a predictor of nursing resilience, through the competencies acquired in performing nursing tasks and knowledge related to practice, as well as communication competencies [11, 14, 20]. In this regard, our study shows that workplace resources facilitate the occupational health of new nurses by promoting the satisfaction of basic psychological needs. More specifically, previous work has indicated that high-quality interpersonal relationships are conducive to communication and trust, thus meeting the specific needs of new nurses [24, 25]. Finally, our results are consistent with the adaptive phases in the trajectory of professional integration that have been established by Baharum et al. [2]. According to these researchers, the first two adaptive phases concern professional adaptation, first in the academic institution and then in the occupational organization, and the third is a social adaptation through the personality of nurses. Thus, as our results show, at the time of career transition, new nurses display their *know-how* to carry out their professional activities, which is consistent with the factors associated with the practice environment.

#### Nursing resilience and its relationship to occupational health and optimal functioning

The study supports the importance of reporting on the developmental (stable or malleable) nature of resilience [67]. The results show that adaptation at the beginning of a nursing career is linked with distinct psychological and professional experiences. More specifically, when associated with the fulfillment of the need for competence, nursing resilience among new nurses appears to be linked to greater energy at work (vigor) and reduced emotional exhaustion, both of which contribute positively to their workplace well-being. In addition, resilience seems to allow new nurses to have a more positive experience that results in a decrease in the occupational turnover intentions. This is particularly important, as nearly 50% of new nurses report intentions to leave their job within the first year of their career [68]. In addition, measuring various resilience outcomes is essential, as Howard Catton, Director General of the International Council of Nurses, emphasizes that the health of nurses will be the most critical determinant of population health in the coming years. In this, we recognize the ambivalence linked to the desire to continue in the nursing profession, or even to leave it prematurely. It is therefore essential to have a global vision in terms of health and functioning. Moreover, our results suggest that the repercussions may not arise from the same underlying dynamics. Remember, for example, that competence and resilience are positively related to vigor and negatively related to emotional exhaustion and the turnover intentions, while autonomy is positively related to affective commitment. By taking into account both positive and negative attitudes of functioning, the results suggest potential avenues for promoting manifestations in terms of well-being and potentially prevent those in terms of ill-being.

#### Basic psychological needs theory: a mini-theory of self-determination theory

The derived model offers a new way of understanding the lived experience of career transition, which allows us to better assess this context and also, in a meaningful way. Building on this, the distinct evaluation of the three basic psychological needs provides further insight. Having already addressed the unique contribution of competence as a lever of resilience, a further contribution of this study lies in the mediating role of the need for autonomy in explaining the affective commitment to the nursing profession at the beginning of a career. Previous results have shown that supporting nursing autonomy is a guarantee of commitment to the profession [69]. In addition, autonomy is linked to competence. More specifically in nursing practice, these psychological needs are related to clinical knowledge and competencies [70]. In the present study, this may even explain the trend observed in the relationship between the need for competence and affective commitment.

For its part, the need for relatedness seems less inclined to be related occupational health at the beginning of a career. It is possible that it is only later in their professional trajectory that nurses assimilate and integrate the norms of the work team [2] However, this finding warrants deeper consideration. When individuals’ needs for autonomy, competence and relatedness are fulfilled, this fosters the internalization of motivation and contributes to the development of adaptive career behaviors [18]. In line with this idea, the findings of the study by Sönmez et al. [18], conducted in the context of nursing education, show that the satisfaction of students’ psychological needs facilitates career adaptability, including the need for relatedness. In this regard, practical experiences (e.g., clinical placements) are structured components of nursing education, that is, supervised and guided learning opportunities. In contrast, immersion in the practice environment at the beginning of a nursing career marks a transition where the new nurse is exposed to the realities of professional practice. Considering that nurses’ basic psychological needs are met within the work environment, a plausible explanation for the absence of any association between the need for relatedness and the outcomes in our study would be the context of the shortage and major changes in the network, while new nurses are exposed to a growing demand for care [71] and are not feeling adequatly competent in all of their role. From the outset, new nurses draw on their adaptive resources by prioritizing their need for competence. In this way, they can demonstrate their professional development through, among other things, the achievement of their professional objectives set from their knowledge and expertise acquired during the training. Our results also highlighted that the need for autonomy seems to be a psychological and motivational experience that is conducive to affective commitment from the beginning of a career. Thus, the positive practices of the referent act as psychosocial and organizational determinants that foster the psychological development of new nurses. While the quality of interpersonal relationships satisfies psychological needs, our findings shed light on their distinct action. Following the example of our derived model, nursing resilience appears to support new nurses in adapting more effectively to their role, largely due to their perceived competence at career start – competence shaped by the quality of interpersonal relationships in the workplace. This highlights nursing resilience as a psychological mechanism that is just as essential as basic psychological needs in the process of adaptation. Moreover, adaptability makes it possible to redesign objectives and resources to better adapt to the lived experience of the workplace [72]. Indeed, through adaptability, the individual is likely to adopt more proactive behaviours and attitudes at work and thus act on his/her health and functioning. Therefore, it seems possible to believe that psychological and adaptive mechanisms could differ according to the context or during other pivotal stages of the professional trajectory [15]. Although unique to each new nurse, the adaptive process is useful at all stages of the career and more importantly, resilience would be a key to adapting to the one-time stressors of nursing work in the early career [16, 72].

### Limitations and prospects for future research

The study proposes new understandings of the links and mechanisms associated between psychological needs and nursing resilience in the relationship between the quality of interpersonal relationships and occupational health and functioning. In addition, it considers both the formal and informal referent. The literature reviewed appears to focus primarily on the role of formal mentors in promoting career retention, in addition to assisting with professional development and ensuring safe and effective care. Nonetheless, our findings indicate that support – irrespective of its structure – is beneficial to the occupational health of new nurses when it fosters adaptive psychological coping mechanisms.

However, some limitations must be named. First, the data is self-reported. There is a risk about the tendency to offer social desirability responses. However, several procedural precautions have been taken to minimize this risk. Data were collected through general data collection as part of the principal investigator’s doctoral continuum. As a result, many of the dissociated variables in this study were measured and mixed with its variables. The measures used in this study were positioned at a distance from each other to divert participants’ attention away from the study variables and thus restrict the tendency of participants from using their previous responses [73]. Moreover, since the majority of the variables studied fall under dimensions such as personal resources or psychological states, self-reference is generally the choice chosen to study these variables [74]. Nevertheless, future studies may use more objective measures for some of the variables studied, including workplace resources. For example, evaluation by the significant referent and observations in the practice environment would improve the validity of the model. In particular, these techniques would make it possible to focus on the attitudes of new nurses through their behaviours, situations and objective facts, rather than on their declaration.

Secondly, we note the poor metrological quality of the resilience measurement instrument. Indeed, the short scale of resilient adaptation did not meet the requirement of measurement reliability. More specifically, internal consistency indicates the extent to which an instrument is capable of accurately measuring the construct it is intended to assess. It reflects the degree to which the items are interrelated and collectively contribute to a unified and reliable measurement of the concept. In this regard, a factor analysis was conducted to examine the structure of the items, and the results indicated that the organization of the instrument was adequate. To our knowledge, there is not validated or dedicated an instrument for measuring *the state of resilience* specifically for the nursing population, which could explain a Cronbach’s alpha below the thresholds typically accepted by the scientific community. Despite the presence of some limitations, the BRCS remains a psychometrically reliable and relevant tool for assessing levels of resilient adaptation. In this perspective, Peterson et al. [75] highlight the value of this measure for the assessment of resilient adaptation. To our knowledge, the internal consistency of this measure is never very high. To the best of our knowledge, there is no study conducted on a nurse, student or working population that has used this measurement tool. However, previous studies of university and general student populations show a variation of 0.59 to 0.78 [75, 76]. Finally, although one of the main findings of our study must be interpreted with caution due to the low internal consistency associated with the concept of resilience, we argue that nursing resilience remains an essential resource for adaptive capacity in the early stages of a nursing career. It is therefore necessary to look into the development of a standardized measurement instrument that will meet the needs of the governance of the health network and researchers, and that accurately reflects nursing resilience within a specific context. In this regard, the start of a nursing career is often tumultuous, and resilience as a resource may be put to the test. Its dynamic nature can influence measurement outcomes, making low scores understandable and expected.

Thirdly, it is not possible to draw conclusions about the cause-and-effect relationships between the variables due to the cross-sectional design. Future studies could validate the proposed model using a longitudinal research design to identify causal factors and determine the validity of the model over time. Moreover, from a temporal and situational perspective, it is possible that new nurses’ perception of the satisfaction of their needs influences their choice of a significant referent. In this regard, at the beginning of their careers, new nurses are looking to develop professionally. To do this, our results show that they must strengthen their need for competence and autonomy.

Fourth, the study was conducted in Quebec, Canada. However, further studies need to be conducted in other Canadian provinces and around the world to confirm the transferability of the results since the limited geographic area may limit the generalizability of the study. In addition, the results of this study are the result of a single governance of the health network. This study could therefore be extended to different health care systems such as the private sector. Also, there is evidence that survey response rates are declining among nurses [1]. This could be explained by the difficult and stressful working conditions that prevailed at the time in the network, which was in the midst of a restructuring. For example, in Quebec, work overload, time constraints, and chronic staff shortages are examples of obstacles encountered at the beginning of a nursing career [4], as is the expansion of professional and family life since the generation that characterizes new nurses prioritizes their life in general over their work [1].

## Practical implications and conclusion

The model presented in this study needs to be validated by additional studies to confirm the results on early nursing career adjustments. Nevertheless, it holds practical significance for enhancing our understanding of psychological coping mechanisms during the early stages of a nursing career. In particular, it could inspire opportunities for professional development and satisfaction, in order to keep new nurses engaged and healthy. In addition, the results of the study highlight the importance of both psychological needs and nursing resilience at the beginning of a career and, more specifically, the need for competence and autonomy on resilience. These results validate Benner’s theory, confirming that it is during the first two years of a career that new nurses work to consolidate their professional competencies to become autonomous. Thus, the perception of competence is most likely important to new nurses in this context. Therefore, targeted interventions aimed at aligning this need with the imperative to reinforce nursing competencies are necessary.

As the mediation mechanism is partial (and not significant for the need for relatedness), this suggests that the quality of interpersonal relationships is a positive practice that can foster engagement among new nurses, as highlighted in leadership theories (abilities to exert a positive influence; [5]) which emphasize the importance of a significant referent figure. This observation addresses the interplay between temporality and affect in shaping interpersonal relationships [59]. In this sense, the first two years of a career are generally devoted to competency development, which justifies, according to Benner, the need for new nurses to work alongside experienced personnel in order to promote their professional integration and possibly a reduction in the daily stressors of practice, as interpersonal relationships are shaped by the occupational context and their perception allows new nurses to rationalize the demands of nursing work [2, 25, 77]. In this way, interpersonal relationships act as a psychological buffer and result from actions and interactions between the referent and the new nurse, his/her health and work. Moreover, it is through interpersonal relationships, where learning takes place, that resources can be developed, strengthened or adapted [77].

In this regard, to prioritize the quality and safety of care, nurses are commonly encouraged to strengthen their leadership for better health care and in their interpersonal and social relationships. From the start of professional training, it would therefore be relevant to focus on the management by and for new nurses and thus that they demonstrate leadership through their *potential* at work and therefore, improve their professional activities in the practice environment. They would thus be able to manage their behaviours to achieve their professional goals at the beginning of their careers. Also, the contribution of exercising the full potential of new nurses on their occupational health would promote their mobilization to facilitate their professional integration and to sustain their commitment to health in the service of those receiving care within the health care system. In this regard, when it comes to nurses’ psychological health at work, the work focuses more on aspects associated with psychological ill-being (e.g., stress, anxiety, burnout) or moral distress (e.g., bullying, psychological violence). For our part, we recognize the urgency of reversing these limiting approaches. In fact, thanks to the dynamics between workplace resources and personal resources, it becomes possible to create a desirable and health-promoting context for psychological well-being at work. In this context, adaptation emerges as a key condition for successful self-management, enabling new nurses, at least to some extent, to ease their transition to a professional practice aligned with both their competencies and their personal needs [55]. Indeed, adaptation, within an environment that fosters well-being, and resilience can contribute to self-management practices [78]. Adaptation then becomes an active lever for professional development and occupational commitment.

In this set of ideas, new nurses will quickly move into management responsibilities (e.g., supervisory activities [coaching and support; preceptorship, mentorship]). In this regard, the Nursing and Midwifery Council [79] documented that nurses must be able to lead, delegate, supervise and stimulate other nurses and health professionals. […] As a graduate, they must be able to think analytically, use problem-solving approaches and evidence in decision-making, track technical advances, and meet future expectations. [Free translation] (p. 4). However, it is necessary to better prepare the student population for leadership and supervisory responsibilities and in this way, adapt and transpose pedagogical strategies to the realities on the ground. To this end, interpersonal relationships, focused on support and listening, and making room for positive leadership practices (e.g., authentic), seem essential to facilitate the transition of new graduates. Formalized programs must be offered to conceptualize trajectories adapted to the needs and even to the work environment [61]. In addition, training could be developed, both for nurses in the network and for new nurses, in order to encourage them to deploy behaviours that are more conducive to the full satisfaction of psychological needs [80]. Strengthening harmonious, empowering, satisfying, and trust-based relationships within care settings, along with enhancing awareness of psychological resources – given their positive association with health and work functioning – constitutes an example of meaningful action. Such behaviours could be particularly useful in the context of career integration, where psychological stress is omnipresent. Indeed, positive and warm relationships help strengthen new nurses’ confidence in their ability to succeed at work, while also fostering a more flexible attitude toward the challenges they face.

In short, the results offer an innovative look at the processes that explain how the quality of interpersonal relationships with the significant referent acts on the motivational and adaptive processes of new nurses in the context of professional integration. By focusing on the quality of support, based on supportive and quality practices, new nurses can achieve a favourable outlook on their occupational health by meeting the needs for autonomy and competence, and their nursing resilience. Positive working relationships allow new nurses to feel confident and adopt a more flexible posture in the face of daily challenges. As these conditions are associated with early career functioning, health care institutions should promote a supportive culture adapted to the needs of new nurses during their integration.

## Data Availability

The data are not publicly available to respect the privacy of research participants.

## Abbreviations

SDT: Self-determination theory
